# Circadian rhythms and mental health

**DOI:** 10.1136/bmjment-2025-301818

**Published:** 2025-06-09

**Authors:** Daniel J Smith, Elise McGlashan, John Gottlieb

**Affiliations:** 1 Division of Psychiatry, University of Edinburgh, Edinburgh, Scotland, UK; 2 School of Psychological Sciences, University of Melbourne, Melbourne, Victoria, Australia; 3 Psychiatry, Chicago Associates, CHICAGO, Illinois, USA

**Keywords:** Adult psychiatry, Sleep

 Well-synchronised circadian rhythms are fundamental for human health and particularly mental health, but patterns of modern living—including, for example, shift work and excess light at night—can cause desynchronisation of rhythms and result in a wide range of adverse mental health outcomes. In recent years, there has been a renaissance of interest in the overlap between sleep/circadian science and mental health, from basic discovery science through to novel chronotherapies and public health policy.^1^ This topic collection brings together five original research papers, two systematic reviews and a perspectives piece, representing an up-to-date overview of current research and innovation in the emerging field of chronopsychiatry.

In a study of almost half a million participants in the UK Biobank cohort, Daudali and colleagues investigated the effects of common genetic variation in the circadian regulator gene *BMAL1*.^2^ Their hypothesis was that circadian disruption (indexed by *BMAL1* polymorphisms) may represent a common mechanism linking psychiatric and cardiometabolic traits. Although they identified associations between *BMAL1* polymorphisms and traits such as body mass index, blood pressure, waist-hip ratio, major depressive disorder, anhedonia, neuroticism and risk-taking, these associations were distinct rather than shared. Clearly, the intersection between the genetics of circadian regulation and mental/physical health comorbidity is complex and represents an important area for future investigation.

Making innovative use of large-scale sleep survey data collected before and during the COVID-19 pandemic (on participants from the Australian Genetics of Depression Study), Shin and colleagues identified a sharp drop in the proportion of participants reporting an ‘optimal’ 6–8 hours of sleep and, further, they found that certain prepandemic characteristics were associated with shifts towards either shorter sleep or longer sleep.^3^ For example, those shifting to longer sleep tended to be younger, with higher distress, an evening chronotype and a higher polygenic score for depression. The authors suggest that we should consider the importance of tailored interventions to improve sleep within different subgroups of people with depressive disorders, particularly during periods of external stress.

In their systematic review,^4^ Aronica and colleagues bring together data on the digital phenotyping of sleep and two important groups of patients: those at ‘clinical high risk’ for schizophrenia and individuals with established schizophrenia spectrum disorders. Their analysis of 18 studies suggests that wrist-based actigraphy may have some utility in identifying sleep abnormalities in these groups compared with controls. However, heterogeneity and biases across the 18 studies were a limitation, highlighting the need to standardise protocols for wrist-based actigraphy, in addition to developing more precise approaches to patient stratification.

Spitschan and colleagues have taken up the challenge to improve standardisation (and harmonisation) in the area of light therapies.^5^ To develop an expert consensus-based, systematic approach to the reporting of light interventions, they conducted a Delphi process. The resulting ENLIGHT (Expert Network on LIGHT Interventions) checklist includes 25 items, representing an important framework for documenting and reporting light interventions in human studies. The potential benefits of adopting the ENLIGHT checklist are obvious: a better understanding of biological mechanisms and more evidence-based, personalised light therapies in the future.

In a similar approach to promoting more consistent and more open data processes, Deeb and colleagues investigated data-sharing practices within the Circadian Mental Health Research Network.^6^ Data sharing is clearly a critical area for research collaboration, reproducibility and transparency. They found that data sharing in this field remains limited, with researchers reporting barriers due to data complexity, privacy considerations, ethical concerns, logistical/technical issues and, unfortunately, an ingrained academic culture that has yet to fully commit to open science practices. The authors offer a number of useful potential solutions, including training on Findable, Accessible, Interoperable, Reusable data principles, data curation services and guidance on ethical issues.

Three of the publications within this topic collection have a more clinical focus. In an interesting pilot study, Crisp and colleagues assessed whether the effectiveness of cognitive bias modification for facial emotion processing in young adults was better if delivered at a time of day synchronised to chronotype.^7^ Their findings may be relevant to the delivery of psychological interventions: facial emotion processing was better when the intervention was synchronised to chronotype (that is, in the evening for late chronotypes and in the morning for early chronotypes). More work on how and when to best deliver online psychological therapies according to chronotype could have important implications for clinical practice.

In another pilot study, Kim and colleagues assessed the feasibility, acceptability and preliminary efficacy of two social rhythm interventions in adolescents and young adults with bipolar disorder.^8^ One approach targeted brain emotion circuitry using self-monitoring/regulation (called BE-SMART-ER) and the other was a social rhythm therapy focused on addressing daily rhythm irregularities (BE-SMART-DR). The authors found preliminary evidence that the interventions were feasible and acceptable and, further, that regularising daily rhythms may enhance the function of emotion regulation brain circuitry and the regulation of emotions. A future larger-scale trial is now warranted.

Finally, Rudisell and colleagues addressed the interesting question of whether the time at which patients with acute agitation are treated with benzodiazepines impacts on clinical response.^1^ They reviewed data from 29 studies (a combination of randomised double-blinded placebo-controlled trials, prospective open-label studies and retrospective reviews). They found that most of the studies did not report the time of day of patient recruitment, but in the four studies that specified time of day, there was some circadian variation in the efficacy of sedation by benzodiazepines. Although somewhat inconclusive, this work should stimulate more detailed research on the potential clinical impact of administering psychotropic medications at different times of the day.

In summary, this topic collection represents an important initial contribution to the nascent field of chronopsychiatry. Collectively, the published papers highlight this as an area of considerable importance for mental health research and future clinical innovation, and we look forward to further development of this field in the years to come.
